# Longitudinal validity of the hemophilia caregiver impact measure

**DOI:** 10.1007/s11136-019-02168-y

**Published:** 2019-03-22

**Authors:** Carolyn E. Schwartz, Jie Zhang, Jun Su

**Affiliations:** 1 DeltaQuest Foundation, Inc., 31 Mitchell Road, Concord, MA 01742 USA; 2 Departments of Medicine and Orthopaedic Surgery, Tufts University Medical School, Boston, MA USA; 3 Bioverativ Therapeutics Inc., Waltham, MA USA

**Keywords:** Hemophilia, Caregiver, Burden, Impact, Longitudinal construct validity, Responsiveness, Hemophilia caregiver impact measure

## Abstract

**Introduction:**

The hemophilia caregiver impact (HCI) measure is a 36-item self-report tool that has documented reliability and validity in a large cross-sectional study, but its longitudinal construct validity is unknown. This study’s objective was to evaluate the responsiveness of the HCI to clinically important change, and to provide interpretation guidelines.

**Methods:**

This web-based study invited 458 hemophilia caregivers involved in the HCI’s validation study to provide follow-up data. Measures included the HCI, and a Likert item querying Global Assessment of Change (GAC) for caregiver burden. Responsiveness was estimated using anchor- and distribution-based methods. The anchor-based method computed the minimally important difference (MID) by computing the mean change separately for those who reported lesser or more caregiver burden on the GAC. The distribution-based method computed the Modified Standardized Response Mean (MSRM) separately for people who reporting reduced or increased burden as compared to the ‘same’ groups.

**Results:**

The study sample included 323 caregivers (71% response rate), with mean follow-up of 21.9 months. The HCI Burden Summary score and all negative-burden subscales but not the Positive Emotions subscale evidenced responsiveness to clinically important differences, showing statistically significant differences by transition group. The MIDs were relatively small mean changes over time (e.g., Burden Summary MID ranged from − 2.2 to 2.6, for reduced versus increased burden), and the MSRMs were small effect sizes. The Burden Summary score was equally sensitive to reduced versus increased burden (MSRM of − 0.32 and 0.35, respectively).

**Conclusions:**

The HCI demonstrated longitudinal construct validity. The HCI shows promise for clinical hemophilia studies as a caregiver-based tool for evaluating treatments.

## Introduction

In the field of clinical hemophilia research, patient-reported outcomes (PROs) can be particularly helpful to complement the clinician-assessed objective outcomes that are important to drug approval (e.g., annualized bleeding rate). Hemophilia is most often a genetic bleeding disorder, usually at birth and can be acquired. The treatment is self-administered or by a caregiver. Therefore, useful person-centered metrics may involve proxy measures (i.e., completed by a parent or other representative of the patient). There are currently several proxy PROs that evaluate the quality-of-life (QOL) of the young patient, including subscales that account specifically for physical, emotional, and social functioning; self-esteem; and stigma [1–3]. In addition to the QOL of the hemophilia patient, recent work has sought to understand the impact of the disease and treatment on family-member caregivers, since the caregiving duties span from birth onward. Research on hemophilia caregivers suggests that caregivers experience burden in physical, emotional, and social domains, as well as impairment of work productivity and financial resources [4–6]. Accordingly, it would be useful to have a reliable and valid measure of hemophilia caregiver burden which also reflects developmental or other changes over time. This study is aimed to examine the responsiveness of the Hemophilia Caregiver Impact (HCI) to clinically important change.

The HCI measure was thus developed and validated [7]. This 36-item caregiver-reported measure assesses the negative and positive personal impacts associated with caring for family members with hemophilia. The measure provides a potentially important metric for the impacts of hemophilia treatments and/or care. Item development used extensive qualitative testing, and item analysis utilized item–response theory methods. The resulting tool has documented internal consistency and test–retest reliability, construct validity, incremental validity, and discriminant validity. Furthermore, the measure proved useful in detecting differences in caregiver burden as a function of the treatment burden in factor-product regimens [8]. What is currently unknown about the HCI is its responsiveness to clinically important change, i.e., its longitudinal construct validity.

While all aspects of developing and validating a patient-reported outcome (PRO) are important, evaluating the longitudinal construct validity of a measure is particularly relevant in clinical research evaluating treatments over time [9]. Responsiveness is an important aspect of validity [10] because its characterization highlights how much change on the PRO score is clinically important, and thus facilitates interpreting the measure over time [11].

Seminal work on clinical significance in PRO research has provided a useful short-list of methods that can be used to assess responsiveness [12–15]. Clinically important change is defined as change noticed by the patient/participant that should be recognized by the clinician as important. Person-reported metrics for defining clinically important change provide distinct information from clinical outcome measures for hemophilia, such as annualized bleeding rate or the presence of target joints. Person-reported metrics may utilize anchor-based or distribution-based methods. Anchor-based methods link change scores to the respondent’s subjective global assessment of change (GAC) over the past few months on the outcome of interest [16, 17]. The GAC allows one to identify how much change (i.e., number of points changed) on a PRO is associated with the respondent’s evaluation of “feeling better” or “feeling worse” with regard to a particular concept. In contrast, distribution-based methods identify clinically important change on the basis of variance estimates, such as one-half standard deviation or effect sizes [18, 19]. Both approaches are useful for providing interpretation guidelines for change over time [20]. The present study thus sought to evaluate the HCI’s longitudinal construct validity.

## Methods

### Design and sample

This longitudinal study included caregivers of people with hemophilia A or B who had who participated in the above-referenced HCI validation study. Study recruitment methods are described in cross-sectional validation article [7]. Eligible participants were fluent in English. Only one caregiver per family unit was allowed to participate. Data were collected at study entry and follow-up about two years later.

#### Procedure

The study protocol was reviewed and approved by the New England Institutional Review Board (NEIRB #14-422). Written informed consent was obtained at baseline. All procedures followed were in accordance with the ethical standards of the responsible committee on human experimentation (institutional and national) and with the Helsinki Declaration of 1975, as revised in 2008. This web-based study was administered using the Health Insurance Portability and Accountability Act of 1996 (HIPAA)-compliant, secure SurveyGizmo engine (http://www.surveygizmo.com). HIPAA is United States legislation that provides data privacy and security provisions for safeguarding medical information.

Study participants were emailed the link to the follow-up survey, and all participants were paid $75 for their fully completed 45-min survey.

### Measures

The HCI measure [7] is a validated 36-item caregiver-reported measure assessing the personal impact associated with caring for people with hemophilia. Responders were asked to complete the survey based on their experience in the past 6-months. The measure has eight domains: seven subscales assess relevant negative aspects of caregiver impact and one subscale assesses positive aspects of caregiving. The negative-impact subscales include: (1) *Practical Impact*, which assesses the impact of ordering supplies, medical appointments, and travel to the hospital; (2) *Symptom Impact*, which assesses the impact of witnessing/suffering from the care-recipient’s pain and caregiver worry and distress related to the hemophilia patient’s symptoms; (3) *Social Impact*, which assesses the impact of hemophilia on the family and spouse/partner relationships; (4) *Physical Impact*, which assesses the impact of hemophilia caregiving on the caregiver’s physical functioning/symptoms; (5) *Emotional Impact*, which assesses the impact of hemophilia caregiving on the caregiver’s emotional functioning/symptoms; (6) *Lifestyle Impact*, which assesses the impact of hemophilia caregiving on the caregiver’s/family’s discretionary activities, such as time for self, exercise, etc.; and (7) *Financial Impact*, which assesses the impact of hemophilia on the family’s financial status and work-related function. The positive-impact subscale comprises *Positive Emotions*, which assesses positive aspects of caregiving related to the sense of purpose and self-worth. Subscale scores are computed as the average of subscale items unless more than one item is missing. The only exception is for the Financial Impact subscale, in which the mean-item score is used with whatever items were available: since three of the five items relate to work-related impacts of hemophilia care, including one that relates to having a spouse or partner, many of our caregivers had missing item data because the item was not applicable to them. Scores are standardized to have a mean of 50 and a standard deviation of 10. Standardized scoring is preferable because it facilitates interpretation: the mean and standard deviation are known so it is easy to understand sample characteristics. Two summary scores can be used: A *Burden Summary score* and a *Positive Emotions score*. The Burden Summary score is created by summing the Practical Impact, Symptom Impact, Social Impact, Physical Impact, Emotional Impact, Financial Impact, and Lifestyle Impact scores. Higher scores on the negative aspects subscales indicate increased burden, whereas higher scores on the Positive Emotions score indicate the caregiver’s perception of personal growth as a result of her/his role as a hemophilia caregiver. For full details about the psychometric characteristics of the HCI, see [7].

In addition to the HCI, we included three Likert-scaled Global Assessment of Change (GAC) items: (a) GAC for caregiver-burden changes; (b) GAC for health changes; and (c) GAC for QOL changes. The caregiver-burden version of this item asked “Compare the demands you feel as a caregiver now with what you experienced when you completed the first survey for this study about 18 months ago. Would you say the caregiving demands are…”. The GAC for health changes asked the respondent to “compare your overall health now…”, whereas the GAC for QOL changes asked the respondent to “compare your quality of life now”. All GAC items contained the same seven response options: much worse (1), somewhat worse (2), a little worse (3), same/no change (4), a little better (5), somewhat better (6), much better (7). We used the GAC as the current standard anchor-based method for assessing responsiveness. Additionally, we included two other GAC items in addition to the GAC_caregiver burden_ of interest to be able to test the construct validity of the items. If the three GAC items were highly correlated, we would not have confidence that the GAC_caregiver burden_ was assessing the specific construct of interest (i.e., clinically meaningful change in caregiver burden per se).

Selected measures from the baseline administration of the study were included to assist in characterizing selection biases in the follow-up sample. These measures, included at baseline but not follow-up, included the *PedsQL Family Impact Module* [21] and the *Work Productivity and Activity Impairment Questionnaire*. High scores on the PedsQL indicate better functioning [22]. High scores on the Work Productivity and Activity Impairment Questionnaire reflect worse impact on time or functioning at work [23]. We also collected demographic, insurance coverage, and medical / treatment information related to all of the hemophilia patient(s) for whom the person was providing care.

### Statistical analysis

Descriptive statistics were used to characterize the study sample. We examined selection bias by using t-tests or Chi-squared tests to compare demographic and patient-reported outcome scores on the sample with baseline data only (attrition sample) and those with baseline and follow-up data (the analytic sample). Correlations among the three GAC items were computed to confirm that respondents were thinking about different concepts when assessing their change since baseline (construct validity). Regression models were computed to confirm that change over time on HCI subscale score was related to GAC on caregiver burden (construct validity).

We then evaluated responsiveness using anchor- and distribution-based methods. The *anchor-based method* computed the minimally important difference (MID) by computing the mean change separately for those who reported lesser or more caregiver burden on the GAC (reduced, same, and increased) [20]. The *distribution-based method* computed the Modified Standardized Response Mean (MSRM) separately for people who reporting reduced or increased burden as compared to the ‘same’ groups. The MSRM is the mean change in scores divided by the standard deviation of change scores in patients defined as stable [24]. We computed this responsiveness index separately for patients who reported reduced versus increased symptoms based on previous prospect-theory-based research suggesting that individuals value gains differently than they value losses [25, 26]. Linear regression models evaluated the relationship between the dependent variables of the HCI subscale scores and Burden Summary at follow-up and the independent variable of transition groups (reduced, same, increased).

Statistical analyses were implemented using Stata 15 [27]. Cohen’s [28] criteria for small (0.20–0.49), medium (0.50–0.79), and large effect sizes (≥ 0.80) for mean comparisons (Cohen’s d) were used to interpret MSRM magnitude.

## Results

### Sample characteristics

The web-based survey was implemented for five months in order to obtain a sample caregiver population of 323 individuals, with a desirable representative response rate of 71% [29]. The follow-up period had a mean of 22 months (SD 1.9), with a range of 18 to 27 months. The follow-up caregiver sample had a mean age of 40.5 years (SD 8.6), and 90% were female (see Table 1). The majority of the sample had some college or higher education, 74% were married, and they were most likely to be the parent of the care recipient, having provided care for a mean of almost 12 years. Caregivers had a mean of 2 children, and 75% were providing care to one person with hemophilia. Most had private health insurance.

**Table 1:** Caregiver sample characteristics

		Have baseline data only (*n* = 135)	Have baseline and follow- up data (*n* = 323)	Test statistic comparing baseline versus FU groups	*P* value
Caregiver age	Mean (SD)	39.71 (8.92)	40.46 (8.56)	− 0.84	0.403
Caregiver gender	Male (%)	16%	10%	2.7046	0.100
	Female (%)	84%	90%		
	Missing (%)	1%	0%		
Caregiver education	High school or less (%)	14%	14%	5.6534	0.130
	Some college (%)	44%	36%		
	College (%)	31%	32%		
	Graduate degree (%)	10%	19%		
Race	American Indian or Alaska Native (%)	4%	2%	1.7903	0.181
	Middle Eastern (%)	1%	1%	Fisher’s exact	0.604
	South Asian (%)	1%	1%	Fisher’s exact	0.640
	Other Asian (%)	3%	3%	Fisher’s exact	0.940
	Black or African American (%)	7%	8%	0.258	0.612
	Native Hawaiian or Pacific Islander (%)	1%	1%	Fisher’s exact	0.844
	Caucasian (%)	81%	81%	0.0283	0.866
Marital status	Never married (%)	10%	7%	Fisher’s exact	0.292
	Married (%)	70%	74%		
	Cohabitation/domestic partnership (%)	5%	4%		
	Separated (%)	1%	4%		
	Divorced (%)	13%	9%		
	Widowed (%)	1%	2%		
Number of children	Mean (SD)	1.79 (1.18)	1.99 (1.20)	− 1.62	0.106
Number of children with hemophilia	0 (%)	23%	12%	Fisher’s exact	**0.035**
	1 (%)	58%	70%		
	2 (%)	16%	15%		
	3 (%)	2%	3%		
	4 (%)	1%	1%		
Number of people caring for with hemophilia	1 (%)	76.30%	75.23%	Fisher’s Exact	0.756
	2 (%)	20%	20.12%		
	3 (%)	2.22%	3.72%		
	4 (%)	1.48%	0.62%		
	5 (%)		0.31%		
Relationship to care recipient	Son (%)	73%	75%	Fisher’s exact	**0.032**
	Daughter (%)	4%	1%		
	Children (%)	10%	16%		
	Brother (%)	0%	0%		
	Other Family Member (%)	8%	6%		
	Multiple Family Members (%)	4%	3%		
Number of years caring for patient	Mean (SD)	12.70 (8.01)	11.67 (7.25)	1.35	0.179
Insurance type^a^	Private (%)	73%	74%	0.006	0.938
	Medicare, Medicaid, CHAMPUS, HIS Supplemental (%)	24%	26.93%	0.5169	0.472
	Does not have insurance (%)	5.19%	4%	0.1575	0.691

Bolded *P* values are statistically significant

^a^Percentages may add up to more than 100 because people can have more than one type of insurance

An examination of differences between the baseline and follow-up samples revealed that they were comparable on most demographic characteristics. There were, however, differences between the two samples such that caregivers who provided both baseline and follow-up data were more likely to be providing caregiving support to one or more than one child with hemophilia (Table 1). A comparison of PRO scores between the two samples revealed that caregivers lost to attrition tended to provide lower scores in terms of the baseline HCI Emotional Impact, and reported reduced functioning on the PedsQL Health-Related QOL, Family Functioning, and Total PedsQL scores (Table 2). There were no differences in work impairment due to health on the WPAI.

**Table 2:** Selection bias in terms of PRO scores at baseline

	Have baseline data only (*n* = 135)	Have baseline and follow-up data (*n* = 323)	Test statistic comparing baseline versus FU groups	*P* value
Baseline PROs				
HCI				
Practical impact	50.44 (9.95)	49.74 (9.96)	0.684	0.49
Symptom impact	49.81 (10.45)	50.04 (9.81)	− 0.2185	0.83
Social impact	51.08 (10.73)	49.47 (9.57)	1.5775	0.12
Physical impact	50.97 (10.66)	49.54 (9.66)	1.3997	0.16
Emotional impact	51.43 (10.66)	49.34 (9.60)	2.0569	**0.04**
Financial impact	50.90 (10.48)	49.53 (9.70)	1.2653	0.21
Positive emotions	50.34 (9.31)	49.90 (10.28)	0.4308	0.67
Lifestyle impact	50.91 (10.17)	49.55 (9.86)	1.3355	0.18
Burden	50.62 (10.28)	49.63 (9.78)	0.8935	0.37
PedsQL				
Parent HRQL	62.12 (22.55)	67.04 (21.63)	− 2.1913	**0.03**
Family functioning score	63.22 (22.98)	69.21 (22.17)	− 2.6019	**0.01**
Total score	60.89 (20.95)	65.72 (20.09)	− 2.317	**0.02**
WPAI				
% Overall work impairment due to health	28.85 (30.40)	24.92 (25.27)	1.2035	0.23

Bolded *P* values are statistically significant

### Correlations among the three GAC items

The three GAC items had small effect-size correlations. The correlations of the GAC_caregiver burden_ with GAC_health_ and GAC_QOL_ were *r* = 0.14 and 0.09 (*P* = 0.01 and 0.11, respectively). These low correlations confirm that respondents were thinking about different concepts when assessing their change since baseline on caregiver burden, health, and QOL. These findings support the construct validity of the GAC_caregiver burden_ item, and thus dispel the concern that the GAC_caregiver burden_ item was tapping the specific construct of interest (i.e., clinically meaningful change).

### Longitudinal construct validity

**Fig. 1:**
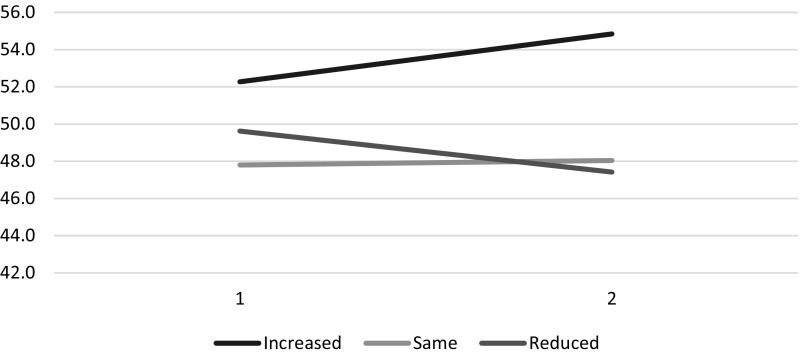
Burden Summary by Global Assessment of Change in Caregiver Burden. This line graph shows means of the Burden Summary score at baseline and follow-up by GAC_caregiver burden_ group. Caregivers who reported worsened burden over follow-up had burden summary scores reflecting increased burden over time. In contrast, those reporting improved burden had declining burden summary scores. Those reporting no change on burden showed no change on the burden summary score

Results of regression models predicting change on HCI subscale and summary scores revealed that the GAC_caregiver burden_ was a significant predictor of change on the following scores: Practical Impact, Social Impact, Physical Impact, Emotional Impact, Financial Impact, and Burden Summary (Table 3). This GAC_caregiver burden_ transition score was not associated with Symptom Impact, Lifestyle Impact, or Positive Emotions change scores. Figure 1 shows mean plots of the Burden Summary at baseline and follow-up by GAC_caregiver burden_ group.

**Table 3:** Minimal important difference and modified standardized response means by transition group

HCI Subscale	Reduced burden (*n* = 69)					No change in burden (*n* = 153)					Increased Burden (*n* = 100)					P*
	MID (Mean Δ)	SD of Δ	MSRM	95% CI		MID (Mean Δ)	SD of Δ	MSRM	95% CI		MID (Mean Δ)	SD of Δ	MSRM	95% CI		
Practical impact	− 2.34	9.39	− **0.25**	− 5.35	− 0.21	0.44	8.49	0.05	− 0.99	1.88	1.77	9.54	0.19	− 0.95	3.61	**0.01**
Symptom impact	− 1.74	8.55	− **0.20**	− 4.38	0.83	0.03	9.40	0.00	− 1.42	1.48	1.68	9.07	0.19	− 0.66	3.96	0.06
Social impact	− 1.70	7.21	− **0.24**	− 4.56	0.14	0.50	8.34	0.06	− 0.81	1.81	2.26	8.72	**0.26**	− 0.33	3.84	**0.01**
Physical impact	− 1.66	7.24	− **0.23**	− 4.28	0.08	0.44	6.82	0.06	− 0.78	1.65	2.48	8.99	**0.28**	0.11	3.97	**0.00**
Emotional impact	− 1.78	7.52	− **0.24**	− 4.72	− 0.06	0.61	8.03	0.08	− 0.68	1.91	2.70	8.74	**0.31**	0.02	4.15	**0.00**
Financial impact	− 1.51	8.10	− 0.19	− 3.49	1.01	− 0.27	8.10	− 0.03	− 1.52	0.99	3.16	7.39	**0.43**	1.44	5.43	**0.00**
Lifestyle impact	− 1.10	8.85	− 0.12	− 3.79	0.69	0.45	7.59	0.06	− 0.79	1.70	1.87	7.51	**0.25**	− 0.57	3.40	0.05
Positive emotions	− 1.47	7.97	− 0.18	− 4.00	1.13	− 0.08	8.91	0.00	− 1.46	1.40	1.45	9.79	0.15	− 0.82	3.73	0.12
*Burden summary*	− 2.20	6.86	− **0.32**	− 4.40	− 0.50	0.24	6.53	0.04	− 0.84	1.33	2.57	7.27	**0.35**	0.60	4.06	**0.00**

Bolded MSRMs are small effect sizes. Bolded MSRM values are statistically significant

*SD* Standard deviation, *MSRM* modified standardized response mean

*P value for group predictor in regression predicting mean change

Table 3 also shows the MID (mean change) and MSRM by GAC_caregiver burden_ group. As expected, the group reporting increased burden showed increased burden subscale and summary scores, and the group reporting reduced burden showed decreases in these scores. These MIDs were relatively small (e.g., Burden Summary MID was − 2.2 to 2.6, for reduced versus increased burden), and the MSRMs were small effect sizes (range |0.20–0.35|). The Burden Summary score was equally sensitive to reduced versus increased burden (MSRM of − 0.32 versus 0.35, respectively).

Different subscales showed more responsiveness in caregivers reporting reduced versus increased burden. For example, Practical Impact and Symptom Impact were more sensitive to change among caregivers reporting lesser burden over time (MSRM − 0.25 and − 0.20 as compared to 0.19 and 0.19, respectively), whereas Financial Impact and Lifestyle Impact were more sensitive to change among caregivers reporting increased burden over time (MSRM − 0.19 and − 0.12 as compared to 0.43 and 0.25, respectively; Table 3). In contrast, Positive Emotions did not reflect change over time by GAC group.

## Discussion

The HCI demonstrated longitudinal construct validity in this study sample. The measure’s summary score was sensitive to subgroup reducing and increasing in self-reported changes in caregiver burden. The detected changes were small effect sizes, suggesting that the HCI summary score is responsive to small but clinically important change.

Four of the negative HCI subscales were differentially responsive to caregivers with reduced versus increased trajectories (i.e., Practical Impact, Symptom Impact, Financial Impact, and Lifestyle Impact). These subscales are important because they highlight salient changes for caregivers with different burden trajectories. In contrast, three subscales and the Burden Summary score were equally responsive to reducing and increasing, suggesting that they capture core or universal aspects of the caregiver-burden construct. These core aspects—physical, social, and emotional impacts—mirror the World Health Organization’s concept of QOL [30]. Based on our findings, the HCI taps core concepts that are universally relevant, as well as some concepts that are more relevant and responsive to positive versus negative changes. Accordingly, each of the negative-burden subscales play an important role in the overall measure’s responsiveness to clinically relevant change.

In contrast, the Positive Emotions subscale was highly stable over time, and did not reflect change reported in the GAC_caregiver burden_. This finding may suggest that Positive Emotions tracks dispositional differences between caregivers (i.e., personality characteristics rather than changeable constructs). The subscale assesses how well the individual is able to use the hemophilia caregiving experience as a growth-inducing experience. Such an ability to transform challenging life events into something positive likely transmits more resilience, critical to Huber’s concept of health as an ability to adapt and to self-manage [31]. Future research might examine how people who score high versus low on the Positive Emotions subscale differ in terms of resilience to personal or caregiving-related health and life challenges. Despite the fact that the Positive Emotions subscale did not demonstrate responsiveness over time, it is a subscale that was developed in response to specific feedback from hemophilia caregivers during early stages of the measure’s development [6, 7]. Caregiver response indicated it was more meaningful when the measure reflects both the positive and the negative aspects of caregiving for someone with hemophilia. Indeed, research on caregivers of other long-term chronically ill populations has demonstrated that positive and negative emotions coexist even at the worse times of loss and challenge, and are necessary for long-term resilience [32–34].

The present study has a notable strength in its relatively low attrition rate and its ability to characterize the few selection biases in the follow-up sample. The limitations of this study should, however, be noted. Because the HCI assesses eight domains and the present study sought to investigate the tool’s responsiveness across domains, there may be some concern that some of our findings capitalize on chance associations due to multiple comparisons. We consider this manuscript to be descriptive not inferential in nature. That is, the focus is on describing the relationships among the HCI subscales and other variables relevant to responsiveness. We thus did not correct for multiple comparisons. If, however, we wished to test specific hypotheses related to caregiver burden, we would deal with the multiple-comparison issue using the two suggested summary scores (Burden Summary and Positive Emotions). Another limitation is that the period of time between baseline and follow-up could result in some measurement errors on the GAC, such as recall bias or implicit theories of change [35]. We believe, however, that such limitations are inherent in all psychometric responsiveness studies, and do not undermine the present study’s findings. Finally, it is possible that the caregiver’s burden could be influenced by changes in their own health or changes in their care recipient’s health. Because the GAC_caregiver burden_ and GAC_health_ were only minimally correlated (*r* = 0.14), it does not seem likely that the caregiver’s health was related to their perceived burden. We are, however, unable to address how their care recipient’s health changes influenced their perceived burden using the present study data. Future research might investigate predictors of caregiver burden, including information about the caregiver and the care recipient’s health over time. Such research might, for example, examine whether caregivers’ own or their care recipient’s health changes influence this burden, and what cognitive and/or behavioral strategies caregivers can use to attenuate their burden over time.

In summary, the HCI shows promise for use in clinical hemophilia studies as a caregiver-based tool for evaluating treatments. Caregiver burden is a significant aspect of hemophilia care Consequently emerging treatments seeking to offer less-frequent dosing interval and better protection should also measure the impacts of these treatments on their respective caregivers. These caregivers are ultimately responsible in many cases to ensure greater continuity for prophylaxis, especially during the pediatric years. The HCI is sensitive to capturing the changes reflected in caregiver burden. It demonstrates both cross-sectional and longitudinal construct validities. In addition to providing a feasible tool to facilitate research, the tool might have applications as a screener for identifying family-member caregivers in need of social work or other supportive interventions in the context of hemophilia clinical practice.
